# Behçet's disease with invasive pulmonary aspergillosis and *Aspergillus auriculatus* infection: A case report

**DOI:** 10.1097/MD.0000000000018938

**Published:** 2020-02-07

**Authors:** Fenfen Sun, Hui Cao, Fan Wang, Guoqiang Cao

**Affiliations:** a Department of Respiratory Disease; b Department of Radiology, Daping Hospital, Army Medical University, Chongqing, China.

**Keywords:** *Aspergillus auriculatus*, Behçet's disease, pulmonary aspergillosis

## Abstract

**Rationale:**

Behçet's disease (BD) is an inflammatory disease that leads to multisystemic immune dysfunction and that involves pulmonary system alterations.

**Patient concerns:**

A 26-year-old woman presented with dull right chest pain for 30 days and intermittent cough with expectoration for 5 days. She had a history of recurrent oral ulcer and constitutional rash 2 months prior.

**Diagnoses:**

The patient was diagnosed with BD complicated by IPA and *Aspergillus auriculatus* infection.

**Interventions:**

The patient was administered itraconazole oral solution (200 mg b.i.d) to treat the fungal infection. After a diagnosis of BD was made, she received 40 mg of methylprednisolone sodium succinate daily for 1 week.

Then, she also received 24 mg of methylprednisolone sodium succinate daily, which was decreased by 2 mg per half month, until the rash had resolved. The patient continued to receive 200 mg Q. D itraconazole orally for 3 months. Thereafter, itraconazole was stopped, while daily oral administration of 10 mg of methylprednisolone sodium succinate was continued.

**Outcomes:**

The rash was observed to resolve, and CT revealed that the lesions in both the right and left lung were reduced. During a telephone follow-up performed after 6 months, the patient stated that no symptoms had recurred during the follow-up period.

**Lessons:**

This case illustrates that for patients with BD, ignoring extrapulmonary symptoms often leads to a delayed diagnosis. Physicians should perform a thorough medical history and physical examination of these patients, as the information obtained in this manner may provide important clues for disease diagnosis and treatment.

## 1 Introduction

Behçet's disease (BD) is an inflammatory disease that leads to chronic, multisystemic immune dysfunction.^[1]^ The manifestations of BD include recurrent ulcers in the oral and genital system and can involve vasculitis, gastrointestinal, central nervous, and pulmonary system symptoms, and articular symptoms. The incidence of BD is higher in “old silk road” regions, especially Northern Turkey (370 per 100,000), than in other areas around the world.^[1–8]^ In China, the prevalence of BD is 14.00 per 100,000 people, similar to Japan and Korea.^[6]^

BD involves pulmonary system alterations^[9,10]^ such as PAAs, PAT, pulmonary infarction, recurrent pneumonia, bronchiolitis obliterans organizing pneumonia, pleurisy, and pulmonary nodules.^[11–15]^ PAAs are the most common cause of death in patients with BD.^[11,16]^ However, few reports have explored the manifestations of BD complicated by microbial infection in lung tissues, such as *Mycobacterium tuberculosis*.^[17]^ Here, we report a case of BD complicated by IPA infection in the pulmonary system and excretion from the left external auditory canal; the patient was successfully treated with systemic corticosteroids and an anti-fungal drug.

## 2 Case report

**Figure 1:**
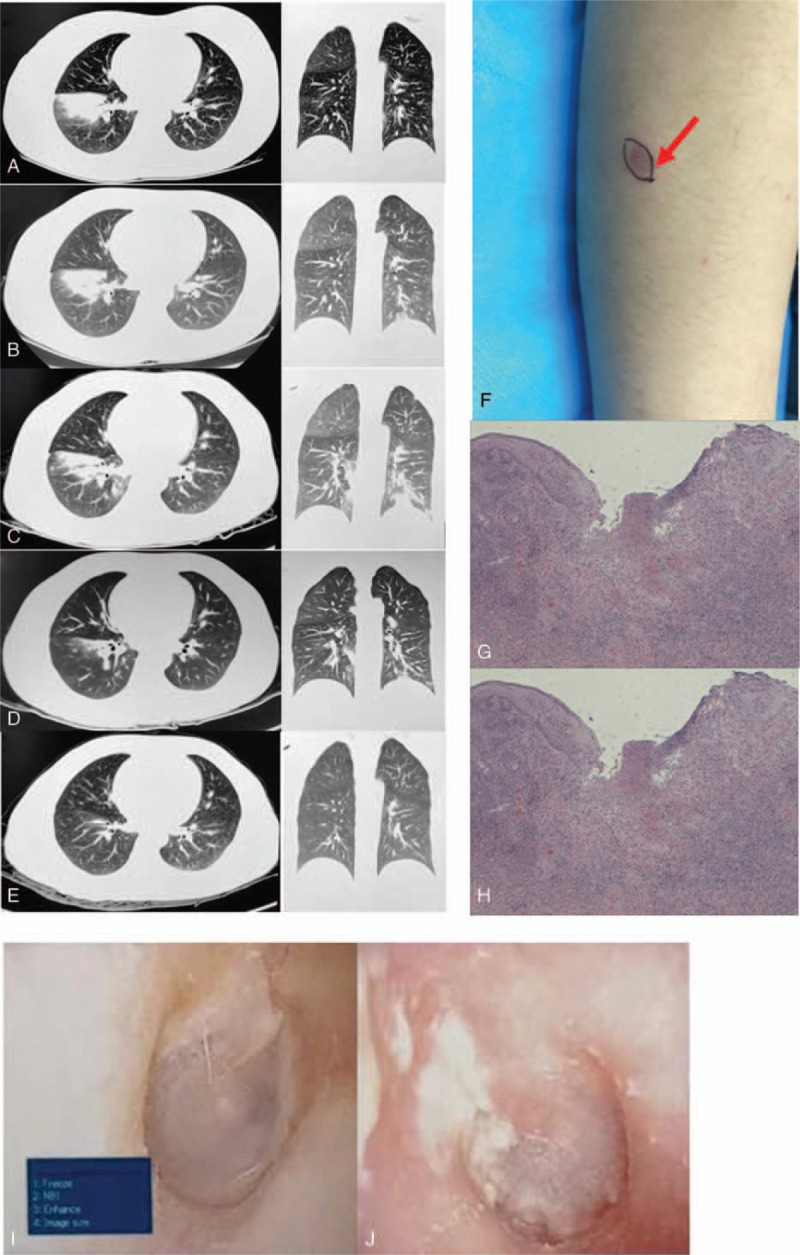
Patient with Behçet's disease complicated by IPA and *Aspergillus auriculatus* infection. A-E, CT scan of the chest on August 24, 2018, August 31, 2018, September 13, 2018, September 26, 2018 and November 1, 2018. F. Rash on the limbs. G and H. Biopsy from genital ulcers (HE stain, ×100). I and J. Otolaryngological examination.

A 26-year-old Chinese female patient presented with dull right chest pain for 30 days and intermittent cough and expectoration for 5 days. She was hospitalized on Aug 24, 2018. CT performed at the outpatient Daping Hospital showed abnormal, lamellar, high-density shadows in the middle and lower lobes of the right lung (Fig. 1A). She had suffered from recurrent oral ulcers and constitutional rashes 2 months prior to hospitalization. There were no adventitious sounds on lung auscultation. Red papules and nodular lesions were observed on the skin of her upper limbs and legs. Additionally, a rash trace was also found on her lower limbs (Fig. 1F). Laboratory examination revealed the following: WBC, 9.68 × 10^9^/L; Neu%, 69.6%, IL-6 and CRP, 48.23 pg/L and 119.6 mg/L, respectively, and ALP and LDH, 152.5 U/L and 709.7 U/L, respectively. Tests for AKA, CCP, ACA, pANCA, cANCA, RNP, dsDNA, (1,3)-β-d-glucan detection, and galactomannan test were unremarkable. T. SPOT and HIV test results were negative.

Based on the above-described condition of the patient and her examination results, the primary diagnosis was pneumonia in the right lower lobe and suspected chronic eczema. The patient received moxifloxacin hydrochloride therapy (400 mg q.d.) for 1 week. Her presenting cough and expectoration were relieved with this treatment; however, her right chest pain worsened. A CT examination showed that while the lamellar high-density shadows were reduced in the middle and lower lobes of the right lung, new lesions were observed in the left lower lung (Fig. 1B, August 31, 2018). She then continued to receive antibacterial therapy for 2 weeks. CT showed that the lesion increased in size, showing enlargement in the left lower lung, while lesions in the right lung were alleviated compared to the result obtained on August31, 2018 (Fig. 1C, September 13, 2018). Given that the patient's lung imaging characteristics did not match the progression of bacterial infection-induced pneumonia, the patient discontinued antibacterial therapy. Importantly, IPA was detected in her BALF. Furthermore, the patient complained that black cerumen was recently excreted from her left external auditory canal. A congested left external auditory canal and tympanic membrane were found on an otolaryngological examination, and *Aspergillus auriculatus* was detected in the excretions of the left external auditory canal (Fig. 1I and J). *Aspergillus niger* was present in cultured secretions obtained from the left external auditory canal. Moreover, the patient informed her doctors that she had been exposed to moldy herbal medicine 3 months ago. Therefore, an itraconazole oral solution (200 mg q.d.) was administered to the patient to treat the fungal infection. Two weeks after the antifungal treatment began, CT examination (Fig. 1D, September 26, 2018) showed that compared to the result in September 13, 2018, the lesions in both the right and left lung had resolved. The patient continued antifungal therapy after discharge.

The patient then stated that she had developed oral ulcers, genital ulcers, and recurrent rash on the upper and lower limbs, eyelid and scalp during the antifungal therapy period (Fig. 1F). In fact, the patient had failed to disclose that she suffered from recurrent oral ulcers, genital ulcers, and skin lesions before hospitalization. A biopsy from genital ulcers revealed superficial necrosis and ulcer formation as well as neutrophil and lymphocyte infiltration in the dermis. Moreover, thrombi were found in several peripheral vessels (Fig. 1 G and H). The patient was ultimately diagnosed with BD complicated by IPA and *Aspergillus auriculatus* infection according to her clinical manifestations and biopsy results.

She was treated with 40 mg of methylprednisolone sodium succinate daily and 200 mg of itraconazole b.i.d. for 1 week. Her clinical manifestations and radiological parameters improved during the treatment period (Fig. 1E, November 1, 2018). Then she received 24 mg of methylprednisolone sodium succinate daily, which was decreased by 2 mg per half month until the rash had resolved. Moreover, the patient received 200 mg itraconazole orally for 3 months. After 3 months of treatment, the lesions in both the right and left lung had completely resolved. Then, itraconazole was discontinued, while she continued to receive a daily oral dose of 10 mg of methylprednisolone sodium succinate. No side effects were reported during the follow-up period. This study was approved by the Medical Ethics Committee of Daping Hospital and the Third Military Medical University, and the patient provided written informed consent.

## 3 Discussion

BD is an inflammatory disease that leads to multisystemic immune dysfunction and vasculitis. It was first reported in 1937, and its characteristics include recurrent oral ulcers, genital ulcers, and uveitis.^[18]^ A recent study suggested that BD should be considered an autoinflammatory disease with immune dysregulation.^[1]^ It is triggered by both the genetic background of the patient (such as HLA-B51) and infectious factors. Several studies have shown that viruses and bacteria play important roles in the development of BD.^[19,20]^ However, few studies have reported on BD patients with fungal infections.

The mechanism of BD with fungal infections is not clear. Several studies have proposed that pathogen infections such as *Mycoplasma fermentans*, *Helicobacter pylori*, Epstein-Barr virus, and hepatitis viruses A, B, C, E, and G can trigger BD occurrence and development.^[1]^ Additionally, other studies have revealed that BD patients exhibit immune system dysfunction.^[21,22]^ Therefore, we speculated that our patient was infected with aspergillosis during an immunocompromised state, and then the aspergillosis infection triggered BD. Meanwhile, the patient's BD aggravated the invasive pulmonary aspergillosis and Aspergillus auriculatus infection.

In this case, a young female was diagnosed with BD complicated by *Aspergillus auriculatus* and IPA infection and exhibited nonspecific pulmonary lesion manifestations. The initial symptoms of the patient were right chest pain, cough, and expectoration; the pulmonary manifestations typical of BD, such as hemoptysis and dyspnea,^[11]^ had not yet been observed. A similar case of pulmonary actinomycosis infection in a young male patient with BD was reported by Scheifer et al^[23]^ Additionally, several reports have shown that pulmonary lesions, including pulmonary artery aneurysm, hemorrhage and infarction, can be identified using conventional chest radiography, CT and spiral CT angiography.^[11,24,25]^ In this case, neither nonspecific lung lesions, including high-density shadow and lymphadenectasis, nor vasculopathy was observed on CT. After antifungal therapy and continuous systemic corticosteroid treatment, the symptoms of the patient, including cough, expectoration and chest pain, as well as ulcers in multiple organs, notably improved, and CT showed that her lung lesions had clearly been reduced.

During the follow-up period, the patient developed multiorgan ulcers and recurrent rashes, and these symptoms did not match the patient's pulmonary imaging results. Under these conditions, obtaining a detailed past medical history and evaluating the extrapulmonary signs of the patient played a key role in providing useful information to guide the respiratory and dermatology physicians towards the final diagnosis of BD complicated by *Aspergillus auriculatus* and IPA infection. Based on this diagnosis, we feel strongly that physicians should perform a thorough medical history and physical examination of these patients, as the information obtained in this manner may provide important clues for disease diagnosis and treatment.

The aim of BD treatment is to control its clinical manifestations, suppress inflammation, elicit an immune response, and prevent the occurrence of secondary organ lesions.^[26]^ Topical and systemic corticosteroids are effective for BD patients with ocular lesions, vascular lesions, etc.^[26,27]^ However, systemic corticosteroids are considered a double-edged sword: On the one hand, they can suppress inflammation and the immune response. On the other hand, they can promote the growth of fungi in vitro,^[28]^ suggesting that under certain circumstances, clinicians should use corticosteroids with caution. In this case, the patient received continuous systemic corticosteroids combined with itraconazole, and the clinical symptoms of the patient were improved; however, we still asked her to continue with routine follow-up in order to monitor her disease progression and adjust her treatment over time.

In conclusion, we report a rare case of a BD patient complicated by IPA and *Aspergillus auriculatus* infection. We found that the patient's detailed medical history and extrapulmonary signs provided useful information to guide the respiratory physician towards a diagnosis of BD complicated by fungal infection.

## Acknowledgments

We thank American Journal Experts (http://www.aje.cn/) for its linguistic assistance during the preparation of this manuscript. We much appreciate professor Huiqiong Chen for the critical comments, good suggestions on this manuscript.

## Author contributions

**Data curation:** Fenfen Sun, Hui Cao.

**Investigation:** Fenfen Sun, Fan Wang.

**Project administration:** Guoqiang Cao.

**Writing – original draft:** Fenfen Sun, Guoqiang Cao.

**Writing – review & editing:** Guoqiang Cao.

Fenfen Sun orcid: 0000-0003-2690-4633.
